# Increased Risk of Group B *Streptococcus* Invasive Infection in HIV-Exposed but Uninfected Infants: A Review of the Evidence and Possible Mechanisms

**DOI:** 10.3389/fimmu.2016.00505

**Published:** 2016-11-16

**Authors:** Nicolas Dauby, Mustapha Chamekh, Pierrette Melin, Amy L. Slogrove, Tessa Goetghebuer

**Affiliations:** ^1^Department of Infectious Diseases, CHU Saint-Pierre, Brussels, Belgium; ^2^Institute for Medical Immunology, Université Libre de Bruxelles (ULB), Gosselies, Belgium; ^3^Department of Clinical Microbiology, National Reference Centre for Group B Streptococci, CHU Sart-Tilman, Université de Liège (ULg), Liège, Belgium; ^4^Department of Paediatrics and Child Health, Division of Paediatric Infectious Diseases, Faculty of Medicine and Health Sciences, Stellenbosch University, Tygerberg, South Africa; ^5^Centre for Infectious Disease and Epidemiologic Research, School of Public Health and Family Medicine, University of Cape Town, Cape Town, South Africa; ^6^Department of Paediatrics, CHU Saint-Pierre, Brussels, Belgium; ^7^Université Libre de Bruxelles (ULB), Brussels, Belgium

**Keywords:** HIV exposed uninfected, Group B *Streptococcus*, newborn, infant, inflammation, breast milk, HIV, pregnancy

## Abstract

Group B *Streptococcus* (GBS) is a major cause of neonatal sepsis and mortality worldwide. Studies from both developed and developing countries have shown that HIV-exposed but uninfected (HEU) infants are at increased risk of infectious morbidity, as compared to HIV-unexposed uninfected infants (HUU). A higher susceptibility to GBS infections has been reported in HEU infants, particularly late-onset diseases and more severe manifestations of GBS diseases. We review here the possible explanations for increased susceptibility to GBS infection. Maternal GBS colonization during pregnancy is a major risk factor for early-onset GBS invasive disease, but colonization rates are not higher in HIV-infected compared to HIV-uninfected pregnant women, while selective colonization with more virulent strains in HIV-infected women is suggested in some studies. Lower serotype-specific GBS maternal antibody transfer and quantitative and qualitative defects of innate immune responses in HEU infants may play a role in the increased risk of GBS invasive disease. The impact of maternal antiretroviral treatment and its consequences on immune activation in HEU newborns are important to study. Maternal immunization presents a promising intervention to reduce GBS burden in the growing HEU population.

## Introduction

Group B *Streptococcus* (GBS) is a commensal Gram-positive coccus, colonizing the gastrointestinal (GI) tract of 10–40% of healthy adults. Classification of GBS is based on capsular polysaccharides (CPs) with 10 distinct serotypes (Ia, Ib, and II–IX). Invasive GBS disease includes meningitis, endocarditis, and urosepsis that usually occur in adults with underlying medical conditions such as diabetes, cancer, or advanced age (1). In neonates, GBS is a leading cause of severe neonatal sepsis and meningitis worldwide and accounts for a significant burden of neonatal morbidity, including long-term sequelae such as poor neurodevelopmental outcome and mortality (2–4). Transmission of GBS from a colonized mother to her newborn can occur vertically before or during labor or horizontally during the neonatal period (2). The clinical spectrum of neonatal GBS disease is usually divided into early-onset disease (EOD) that occurs between birth and the sixth day of life and late-onset disease (LOD) that occurs between 7 and 90 days of life.

Risk factors for invasive GBS disease in early life include both maternal and infant parameters. Maternal GBS colonization during the last weeks of pregnancy is a common risk factor for both EOD (2) and LOD (5). In the 1980s, clinical trials demonstrated that GBS EOD might be prevented by intravenous antimicrobial prophylaxis with β-lactams administered during labor and delivery to women who are colonized by GBS (6). These observations have motivated the screening for GBS carriage in late pregnancy and the administration of antibiotic prophylaxis during labor to mothers with a positive GBS culture (culture-based screening) (7, 8). Other maternal and obstetric risk factors for EOD include GBS maternal bacteriuria during the current pregnancy, intrapartum fever, prolonged rupture of membranes, and preterm labor. Risk factors in neonates are less well characterized and mainly include prematurity and low levels of capsular type specific IgG (2, 9).

Recently, accumulating evidence indicates that HIV-exposed but uninfected (HEU) infants suffer from higher infectious morbidity with more severe infections and more infection-related hospitalizations (10). Some studies have shown a correlation between advanced maternal HIV infection and infectious morbidity in HEU infants (11, 12), one in France showing that the risk of severe bacterial infection, including GBS, was higher when maternal CD4 count was lower than 350 cells/mm^3^.

A higher susceptibility for GBS invasive disease in HEU infants has been observed in both Europe and Southern Africa (4, 12–14).

The burden of GBS invasive disease is greater in low-income countries, notably in sub-Saharan Africa (3) where HIV infection prevalence in pregnant women can reach up to 40% (15, 16). Thus, the increased incidence of GBS disease in HIV-exposed infants has large global neonatal public health implications. Herein, we review the clinico-epidemiological studies supporting an increased susceptibility of HEU infants to GBS invasive disease along with the maternal and infant factors potentially contributing to this increased susceptibility and discuss possible interventions to reduce this burden.

## Higher Risk of GBS Invasive Disease in HIV-exposed Infants: Evidence from Epidemiologic and Clinical Studies

While different studies from distinct parts of the world indicate that HEU infants have an increased risk for severe infections (10, 12, 17–20), four studies (two in Western Europe and two in South Africa) suggest an increased risk specifically for GBS invasive disease in infants born to HIV-infected women. A large multicentre retrospective study performed in France between 2002 and 2010 found an elevated hazard ratio of 2.7 for infection by encapsulated bacteria (including GBS) in infants born to mothers with low CD4 count; however, this study did not include a HUU group. Three studies specifically assessed GBS risk in HIV-exposed infants and found an increased risk for GBS invasive disease: two separate studies from South Africa and one from Belgium (summarized in Table 1). In the two South African studies, HIV infection could not be systematically excluded in HIV-exposed neonates. However, in the recent study by Dangor et al. (4) mother-to-child HIV transmission has fallen below 3% in South Africa, making HIV infection least likely to be the primary cause of the increased GBS risk (21).

**Table 1:** **Summary of studies that assessed the risk of GBS invasive disease in HEU infants**.

Reference	*N*	HUU control group	Design	Location	Period	GBS disease risk in HIV-exposed compared to HIV-unexposed
Epalza et al (13)	20,480	Yes	Monocentric retrospective	Belgium	2001–2008	RR = 19.6 for all GBS infection
						Higher severity, higher rate of LOD
Cutland et al. (14)	372	Yes	Monocentric prospective	South Africa	2004–2008	Higher IRR = 2.25 (95% CI 1.84–2.76) for invasive disease
						Higher IRR = 1.69 (95% CI 1.28–2.24) for EOD
						Higher IRR = 3.18 (95% CI 2.34–4.36) for LOD
						Higher RR for both bacteremia (RR = 1.83; 95% CI 1.40–2.39) and meningitis (RR = 3.05; 95% CI 2.20–4.25)
Dangor et al. (4)	122	Yes	Multicentre prospective	South Africa	2012–2014	Higher IRR = 3.40 (95% CI 2.29–4.85) for invasive disease
						Similar IRR for EOD
						Higher IRR = 4.67 (95% CI 2.24–9.74) for LOD
						Higher odds of LOD (OR = 3.5; 95% CI 1.53–8.09) and meningitis (OR = 6.85; 95% CI 2.64–18.31)

*HEU, HIV-exposed uninfected infants; HUU, HIV-unexposed uninfected infants; GBS, Group B Streptococcus; EOD, early-onset disease; LOD, late-onset disease; RR, relative risk; IRR, incidence risk ratio; OR, odds ratio*.

Importantly, HIV-exposed infants seem to suffer from a distinct pattern of invasive GBS disease. Indeed, these three studies consistently showed increased risk of LOD (4, 13, 14). GBS meningitis, a severe manifestation of GBS invasive disease, was also more likely in HIV-exposed infants in the two South African studies (4, 14). Similarly, the Belgian study observed a greater severity of disease in HEU compared to HUU infants (13).

Serotype III strains are the main cause of LOD and meningitis (22). Only one study specifically assessed serotype distribution between HEU and HUU infants and did not find any difference according to HIV exposure; serotypes Ia and III were the most prevalent in both groups (14).

## Maternal GBS Colonization in HIV-infected Pregnant Women

Maternal vaginal colonization by GBS in late pregnancy or at delivery is the main factor associated with both EOD and LOD (23). This link has motivated the recommendation of universal antenatal screening for GBS at 35–37 weeks of gestation and the administration of intrapartum antibiotic prophylaxis (IAP) in case of positive screening. This strategy has been associated with a significantly decreased incidence of EOD but has no impact on the incidence of LOD (9).

Table 2 summarizes the studies reporting GBS carriage prevalence in HIV-positive pregnant women. Seven studies included a control group of HIV-uninfected pregnant women; among them, five reported a similar prevalence in both groups and two studies found a lower prevalence in HIV-infected women.

**Table 2:** **Summary of the studies assessing carriage of GBS’ prevalence in HIV-infected pregnant women**.

Reference	Location	Design	Controls/HIV+	GBS prevalence		*P* value
				HIV− (%)	HIV+ (%)	
Shah et al. (73)	USA	Retrospective	1947/90	26.0	32.2	0.2
Joao et al. (74)	Brazil	Cross-sectional	No control group/158	NA	31	NA
Gray et al. (25)	Malawi	Cross-sectional	1454/402	21.7	19.4	0.4
Cutland et al. (24)	South Africa	Retrospective	1346/1346	23	17	0.0002
El Beitune et al. (75)	Brazil	Prospective	106/101	14.0	19.8	0.28
Mavenyengwa et al. (76)	Zimbabwe	Prospective	249/88	43.1	40.0	NA
Matee et al. (77)	Tanzania	Cross-sectional	276/24	24.3	8.3	0.08
Dangor et al. (32)	South Africa	Cross-sectional	81/83	27.2	32.5	0.4

A PubMed search was performed using the following MeSH terms: “Streptococcus agalactiae,” “pregnancy” and “HIV.”

Most studies have included low numbers of HIV-infected pregnant women. Cutland et al. performed the largest study published so far including more than 5000 pregnant women, of which 1347 were HIV-infected pregnant women in South Africa. This study found a significantly lower prevalence of GBS carriage in HIV-infected compared to HIV-uninfected pregnant women (17 vs. 23%; *P* = 0.0002) (24).

Interestingly, in another large cross-sectional study in Malawi, GBS carriage was associated with CD4 cell count (25); the proportion of women colonized with GBS was significantly higher in HIV-infected women with a CD4 cell count higher than 500 cells/mm^3^ when compared to women with a CD4 cell count lower than 200 cells/mm^3^. However, GBS prevalence in HIV-infected women with high CD4 count (28.2%) was not significantly higher than in HIV-uninfected women (21.7%). The increased colonization rate in HIV-infected women with high CD4 cell count might be biased by the presence of other risk factors for GBS colonization like diabetes or obesity (23), which are expected to be found more often in women with higher CD4 cell counts (26) and were not taken into account. On the other hand, HIV-infected women with low CD4 cell count are known to have increased prevalence of bacterial vaginosis that could compete with GBS and are more likely to take cotrimoxazole prophylaxis resulting in lower GBS carriage rates (27–29). In the Cutland et al.’s study, CD4 cell count was only available in a limited proportion of HIV-infected women but the majority had a CD4 count >350/mm^3^ (24).

In summary, HIV infection is not associated with higher prevalence of GBS colonization rate in pregnant women. On the contrary, recent evidence indicates that HIV infection might even be associated with lower GBS colonization rate, particularly in pregnant women with low CD4 counts.

The hypervirulent clone ST-17, which belongs to serotype III strains, accounts for the majority of GBS meningitis and LOD sepsis (22). Antibiotic use has been shown to be critical in the selection of virulent GBS strains (30). HIV-infected women are exposed more frequently to antibiotics for therapeutic or prophylactic use (31). Limited data exist regarding the GBS serotype distribution in HIV-infected mothers. In a South African study on 164 pregnant women (83 HIV-infected and 81 HIV-uninfected), serotype III predominated in HIV-infected women (11/27, 40.7% of all serotypes vs. 13.6% in HIV-uninfected women), while serotype Ia was predominantly found in HIV-uninfected women (13/22, 59.1% of all serotypes vs. 29.6% in HIV-infected women) (32). In a recently published study performed in Kenya, while HIV infection was associated with lower risk of GBS colonization rate, HIV-infected women had higher risk of being colonized with the hypervirulent clone CC17. This risk was even higher for those taking co-trimoxazole prophylaxis (29). This predominance of serotype III in HIV-infected women could explain the higher incidence of GBS LOD in HIV-exposed infants (4, 13, 14) but needs confirmation in larger cohorts. The serotypes associated with invasive GBS disease in HEU infants in developed countries have not been characterized.

## Transfer of GBS-specific Maternal Antibodies

Maternal antibodies are actively transferred to the fetus through the placental Fc receptors during the third trimester of pregnancy. Levels of maternal IgG and cord blood IgG are strongly correlated (33). Strong evidence indicates that transplacentally transferred CP-specific IgG protects infants against invasive GBS disease. Indeed, low levels of capsular IgG in mother and newborn are associated with higher risk of EOD and LOD associated with different serotypes (34, 35).

The transfer of maternal IgG against pathogens and vaccine-specific antigens (17, 36–39) is known to be diminished in HEU infants. One of the causes might be maternal hypergammaglobulinemia associated with chronic HIV infection that could compete with specific maternal IgG at the level of the FcRn (33). Uncontrolled HIV infection and the use of combination antiretroviral therapy (cART) are both associated with preterm delivery (40–42), which results in lower concentrations of maternally derived antibodies.

A recent study performed in South Africa assessed the levels of IgG to both capsular and surface proteins in 164 HIV-infected (43% of whom were on cART) and uninfected mother-newborn dyads. Median capsular antibody concentrations were found to be lower for serotypes Ib and V in HIV-infected pregnant women compared to controls and for all serotypes studied (Ia, Ib, III, and V) in HIV-exposed compared to HUU newborns. The cord-maternal ratio was 37.4 and 32.5% lower for serotypes Ia and III, respectively, in HIV-infected mother-newborn dyads. No correlation was found between maternal CD4 cell count (median 423 cells/mm^3^) and transfer of capsular antibodies (32). Unfortunately, the impact of maternal hypergammaglobulinemia, that is expected to decrease after cART initiation (43), was not investigated.

In a smaller study performed in South Africa, capsular antibody concentration was also found to be lower in both HIV-infected pregnant women and their newborns, when compared to HIV-uninfected women and their HUU newborns. Importantly, HEU infants at 16 weeks of age still had lower concentrations of GBS-specific antibodies against all serotypes studied. Moreover, HEU newborns had lower concentrations of antibody-mediated complement deposition on all GBS serotypes suggesting different functionality of GBS-specific antibody (44). Antibody, along with complement, contributes to opsonophagocytosis of GBS strains (45, 46). The opsonophagocytic (OPA) activity of GBS serotype-specific antibodies has been shown to inversely correlate with colonization in pregnancy (47).

In summary, HEU infants have lower levels of maternally derived GBS-specific antibodies with potentially lower OPA activity contributing to an increased risk of postnatal colonization and of EOD and LOD.

## Impact of Breastfeeding Practice

Breastfeeding plays a critical role in the protection against infectious diseases of the infants. Transfer of pathogen-specific IgA and IgG originating from mucosal and milk B cells complements IgG transferred during pregnancy (48).

Two studies provide evidence that both GBS-specific IgG and IgA can be detected in breast milk. Edwards et al. (49) studied nine women 2 months after delivery with sera levels of type III-specific capsular IgG above the correlate of protection against EOD (1 μg/ml). Serotype III capsular IgG were found in breast milk in 3/9 women, those with the highest serum concentrations. IgA was also found in 6/9 women. Similarly, Lagergård et al. measured a prevalence of 63% GBS-specific IgA in milk samples from 70 women (50).

Breast milk avoidance is generally recommended in HIV-infected mothers in high-income countries but not in developing countries (51) and avoidance of breastfeeding in HEU infants might contribute to decreased IgG and IgA GBS-specific levels in HEU infants at the level of GI tract. Colonization of the GI tract of infants is the first step before invasive disease. An animal model has shown that maternal breast milk antibodies protect against penetration of commensal bacteria through the intestinal mucosa (52). In an animal model of GBS infection, suckling animals exposed to maternal antibodies were protected against an oral challenge with serotype III GBS strains (53). Breast milk thus provides levels of GBS-specific IgG and IgA that potentially limit GBS invasion of the intestinal mucosa and subsequent hematogenous spread.

Conversely, as reviewed recently (54), breast milk could also represent a vector of GBS transmission during the postnatal period. Breast milk feeding has been significantly associated with GBS colonization in Gambian infants after birth (55). The role of breastfeeding by HIV-infected women in transmission of GBS and development of sepsis in HEU children have not been evaluated yet.

## Impact of HEU Infants’ Immunity

The innate immunity plays a pivotal role against GBS infection not only by its direct effect against the pathogen at early stages of infection but also by shaping subsequent adaptive immune responses. Innate immune response against extracellular bacteria such as GBS involves various cells including neutrophils and antigen-presenting cells (monocytes and dendritic cells among others). Neutrophils play a fundamental role in the elimination of invading bacteria through phagocytosis and microbial killing (56). In newborns and in preterm infants, neutrophils display qualitative defects including impaired migration to inflammed sites and lower production of antimicrobial peptides such as the bactericidal/permeability-increasing protein (57). Although the functionality of neutrophils in HEU newborns has not been studied, various studies have reported prolonged neutropenia in HEU infants exposed to nucleoside reverse transcriptase inhibitors (NRTI) that could persist up to 24 months of age (58–60).

Monocytes and dendritic cells play an important role in the initiation of the innate immune response against GBS and are activated through recognition of pathogen-associated molecular patterns (PAMPs) by toll-like receptors (TLRs) (61, 62). Upon contact with GBS, innate cells produce high amounts of TNF-α that is one of the major mediators of bacterial clearance and also of the immunopathology of GBS invasive disease (63, 64).

The exposure of HEU infants to maternal HIV-derived products and to a proinflammatory intrauterine environment result in modifications of the phenotype and function of neonatal innate immune cells (17, 65, 66). Single-cell analysis performed in South African infants demonstrated significant differences at early life periods in the inflammatory response of innate cells between HEU and HUU infants that were restricted to certain types of TLR stimulation (67). HEU infant-derived monocytes produced more TNF-α and IL-6 than did HUU infant cells upon their stimulation with bacterial PAMPs, LPS, and PAM but not with single strand RNA (R848). *In vitro* experiments indicate that the production of TNF-α by monocytes/macrophages upon interaction with GBS depends upon bacterial single-strand RNA recognition rather than peptidoglycan (68). It remains to be established how HEU infant innate cells would respond to multiple stimuli induced by whole bacteria instead of TLR individual stimulation.

When tightly controlled, the inflammatory response can be host beneficial through promoting an efficient immune response against the pathogen. Yet, an excessive inflammatory response, which is frequently observed in neonatal GBS infection, can lead to fulminant septic shock and a poor outcome (62). The ratio of proinflammatory vs. anti-inflammatory cytokines, such as IL-10, is thus critical for the outcome of GBS infection. IL-10, the production of which is induced by GBS glycerinaldehyde-3-phosphate-dehydrogenase enzyme, has a dual role in the susceptibility to GBS and disease severity (62). On the one hand, IL-10 may be regarded as beneficial for the host through the control of the excessive inflammatory response that can be damaging for host tissues (69). On the other hand, the immunosuppressive effect of IL-10 may be deleterious. A mice study has shown that TLR2-induced IL-10 production increases GBS susceptibility by limiting the recruitment of neutrophils to infected tissues during neonatal bacteria sepsis (70). In a clinical study comparing HIV-infected pregnant women either receiving cART or not, it has been shown that polyclonal stimulation of cord blood mononuclear cells from HEU newborns of mothers with a detectable viral load had higher proinflammatory vs. IL-10 ratios when compared to HEU newborns of mothers with an undetectable viral load (71). This suggests that the exposure of neonates to maternal immune activation favors a proinflammatory state.

In summary, both exposure to cART and chronic maternal immune activation induce quantitative and qualitative defects of innate immune cells. These abnormalities may participate not only in the increased susceptibility to GBS invasive disease but also in the increased severity observed in clinical studies (13, 14), possibly as a consequence of more highly activated innate immune responses.

## Conclusion and Perspectives

There is consistent evidence from clinical and epidemiological studies that

(1) HIV-exposed and HEU infants have an increased susceptibility to GBS invasive disease compared to HUU infants;
(2) GBS disease in HIV-exposed infants presents with more severe manifestations such as meningitis than in HUU infants; and
(3) HIV-exposed infants have a substantially elevated risk for LOD that is not associated with a higher prevalence of GBS carriage in HIV-infected women.

We thus hypothesize that this increased susceptibility to severe GBS disease results from accumulating factors including (1) lower concentrations of GBS-specific capsular antibodies with potentially lower OPA activity that promotes GBS colonization during the postnatal period and increases the risk of progression from colonization to invasive disease (32, 34, 44); (2) an activated and “hyper-responding” HEU infant immune system that generally favors immunopathology (62, 67, 71).

Consequently, interventions could be proposed to lower the risk and the severity of invasive GBS disease in HEU infants (Table 3). These include universal maternal cART, potential maternal GBS vaccination, and possibly IAP.

**Table 3:** **Summary of proposed interventions to decrease GBS burden in HEU infants and their impact and limitations**.

Intervention	Impact	Limitations
Intrapartum antibiotic prophylaxis	Decrease of EOD incidence (7)	No impact on LOD incidence (9)
Maternal immunization with a trivalent vaccine (Ia, Ib, III)	Lower maternal and postnatal colonization rate (47)	Only cover specific strains (78)
	Lower incidence of invasive disease (34)	Lower response to vaccine in HIV-infected women (72)
Breastfeeding promotion	IgG/IgA at the level of GI tract can decrease GBS invasion (49, 50, 53)	GBS transmission through breast milk (54)
Antiretroviral treatment before conception with immune restoration	Decreased neonatal immune activation (71)	Higher rate of premature birth associated with some regimens (41)
Less toxic NRTI or NRTI sparing regimen	Preservation of neutrophils count (58–60)	

Low maternal CD4 count has been associated with severe infections in HEU infants, including GBS (11, 12), suggesting that cART initiation before conception and the subsequent immune restoration could prevent deleterious consequences of HIV exposure, likely through control of maternal inflammation (39). Maternal immunization during pregnancy appears as an efficient strategy to provide adequate levels of capsular specific antibodies in the neonate. A phase II trial on the use of trivalent glycoconjugate GBS vaccine (against serotypes Ia, Ib, and III) in HIV-infected and uninfected pregnant women was recently published (72). The concentrations of maternal antibodies were lower in HIV-infected women though, irrespective of the CD4 count. Of note is that the difference between HIV-infected and uninfected women was greatest for serotype III-specific antibody, serotype III being the major cause of LOD (23). Correlates of protection remain to be established to further validate the impact of this vaccine in the protection of HEU infants against GBS diseases. However, more studies are needed to assess whether or not HIV-infected pregnant women are colonized by specific strains (29, 32), which would impact vaccine policy. Evidence suggests that HEU infants are more likely to suffer from LOD (4, 13, 14); therefore, IAP that results in significant reductions in EOD will not contribute to a substantial decrease of GBS burden in this population (9). Studies in Europe [i.e., Belgium and France (12, 13)] have reported increased invasive GBS disease in HEU infants despite an IAP strategy being in place.

Finally, HEU infants represent a model to decipher host immune responses toward GBS in early life. Studies about the innate response in HEU infants correlated with clinical outcomes could provide new information about mechanisms of GBS invasive disease immunopathology.

## Author Contributions

ND reviewed the literature, drafted the review, and prepared the tables. MC drafted the paragraph on innate immunity and edited the review. PM reviewed and edited the review. AS reviewed and edited the review. TG initiated, supervised, reviewed, and edited the review.

## Conflict of Interest Statement

The authors declare that the research was conducted in the absence of any commercial or financial relationships that could be construed as a potential conflict of interest. The reviewer HJ declared a shared affiliation, though no other collaboration, with one of the authors AS to the handling Editor, who ensured that the process nevertheless met the standards of a fair and objective review.
